# Hall and field-effect mobilities in few layered *p*-WSe_2_ field-effect transistors

**DOI:** 10.1038/srep08979

**Published:** 2015-03-11

**Authors:** N. R. Pradhan, D. Rhodes, S. Memaran, J. M. Poumirol, D. Smirnov, S. Talapatra, S. Feng, N. Perea-Lopez, A. L. Elias, M. Terrones, P. M. Ajayan, L. Balicas

**Affiliations:** 1 National High Magnetic Field Laboratory, Florida State University, Tallahassee-FL 32310, USA; 2 Physics Department, Sourthern Illinois University, Carbondale-IL 62901-4401, USA; 3 Department of Physics, Department of Materials Science and Engineering and Materials Research Institute, The Pennsylvania State University, University Park, PA 16802, USA; 4 Department of Mechanical Engineering and Materials Science, Rice University, Houston, TX 77005 USA

## Abstract

Here, we present a temperature (*T*) dependent comparison between field-effect and Hall mobilities in field-effect transistors based on few-layered WSe_2_ exfoliated onto SiO_2_. *Without* dielectric engineering and beyond a *T*-dependent threshold gate-voltage, we observe maximum hole mobilities approaching 350 cm^2^/Vs at *T* = 300 K. The hole Hall mobility reaches a maximum value of 650 cm^2^/Vs as *T* is lowered below ~150 K, indicating that insofar WSe_2_-based field-effect transistors (FETs) display the largest Hall mobilities among the transition metal dichalcogenides. The gate capacitance, as extracted from the Hall-effect, reveals the presence of spurious charges in the channel, while the two-terminal sheet resistivity displays two-dimensional variable-range hopping behavior, indicating carrier localization induced by disorder at the interface between WSe_2_ and SiO_2_. We argue that improvements in the fabrication protocols as, for example, the use of a substrate free of dangling bonds are likely to produce WSe_2_-based FETs displaying higher room temperature mobilities, i.e. approaching those of *p*-doped Si, which would make it a suitable candidate for high performance opto-electronics.

Field-effect transistors (FETs) based on exfoliated transition-metal dichalcogenides (TMDs)^1^,^2^,^3^,^4^ were shown to be promising as low-power switching devices and therefore as potential components for high-resolution liquid crystal and organic light-emitting diode displays, particularly in their multi-layered form^5^. Bulk transition metal dichalcogenides (TMD) crystallize in the “2*H*'' or trigonal prismatic structure (space group *P*6_3_/*mmc*), in which each transition metal is surrounded by six chalcogenide atoms defining two triangular prims. Extended planes, which are weakly or van der Waals coupled, result from the tessellation of this basic unit. Contiguous planes are shifted with respect to one another (along both the **a-** and the **b**-axis), therefore the unit cell is composed of two planes with a transition metal dependent inter-layer distance *c*. The covalently bonded layers are expected to display high crystallinity, although as in graphite/graphene, one can expect crystallographic mosaicity between planes stacked along the *c*-axis. Similarly to graphite, compounds such as MoS_2_, WS_2_, etc., are exfoliable layered materials characterized by a weak inter-planar van der Waals coupling^1^. In contrast to graphene, they exhibit indirect band gaps ranging from ~1 to ~2 eV which become direct in single atomic-layers^6^, making them promising candidates for applications.

Early studies^7^ on field-effect transistors (FETs) based on bulk WSe_2_ single-crystals using parylene as the gate dielectric, revealed room temperature field-effect mobilities approaching those of *p*-Si^8^ but with a small current ON/OFF ratio. Subsequent investigations^9^ on mechanically exfoliated MoS_2_ flakes composed of tenths of atomic layers and SiO_2_ as the gate dielectrics, revealed considerably lower mobilities (10–50 cm^2^/Vs), suggesting either a remarkable difference in mobilities between MoS_2_ and WSe_2_ or that an inadequate choice of gate dielectrics can hinder their performance. More recently^10^, it was suggested that field-effect carrier mobilities surpassing 1000 cm^2^/Vs could be achieved in dual gated, single-layer MoS_2_ FETs through the use of a top gate composed of a high-κ dielectric such as HfO_2_. Nevertheless, it was argued that this is an overestimated mobility value due to the capacitive coupling between both top and back gates^11^, a fact that is supported by subsequent reports of much smaller mobilities in similar devices when the gate capacitance is extracted from a Hall-effect study^12^,^13^. It was also recently argued that remote phonons from dielectric layers such as HfO_2_, can limit carrier mobility and would require the use of an interfacial layer to absorb most of the vibrational energy^14^. Nevertheless, these observations already led to the development of integrated circuits based on single^15^- and on bi-layered^16^ MoS_2_. Recent studies in both single- and double- layered MoS_2_ revealed Hall mobilities which increase strongly with gate voltage, saturating at maximum values between ~200 and ~375 cm^2^/Vs at low temperatures^17^. In multi-layered MoS_2_ the Hall mobility has been found to increase from ~175 cm^2^/Vs at 60 K to 311 cm^2^/Vs at *T* = 1 K at back-gate voltages as large as 100 V^18^. However, marked discrepancies were reported between the measured field-effect and the Hall mobilities^17^, which at the light of Refs. 11,12,13 could be attributed to underestimated values for the gate capacitances.

Similarly to past research on graphene, much of the current effort on TMD-based FETs is focused on understanding the role played by the substrates, annealing conditions and the work functions of the metallic contacts. For example, it was recently argued that most of the above quoted mobilities are determined by the Schottky barriers at the level of the current contacts which limits the current-density that can be extracted from these transistors. The authors of Ref. 19 argue that small Schottky barriers, and therefore nearly Ohmic contacts in TMD based FETs, can only be achieved through the use of metals with small work functions such as Sc. Furthermore, due to the detrimental role played by the SiO_2_ substrates, Ref. 19 finds that the highest mobilities (~175 cm^2^/Vs) can be achieved in FETs built on ~10 nm (~15 layers) thick flakes. Thickness dependent mobilities were also recently reported for MoS_2_ based transistors using polymethyl methacrylate (PMMA) as the gate dielectrics^20^. High performance TMD-based FETs have been claimed to have the potential to make a major impact in low power optoelectronics^5^,^21^,^22^,^23^. Here, to evaluate this assertion, we study and compare field-effect and Hall mobilities in field-effect transistors based on few-layered WSe_2_ exfoliated onto SiO_2_, finding that they can display room temperature hole-mobilities approaching those of hole-doped Si^8^ with a large ON to OFF ratio (>10^6^) and sharp subthreshold swings (ranging from 250 and 140 mV per decade). This observation is remarkable given that i) carrier mobility is expected to be limited by the scattering from intrinsic^24^ as well as substrate phonons, ii) the Schottky barriers at the contacts have yet to be optimized, and as we show iii) the presence of charge traps and disorder at the interface between WSe_2_ and SiO_2_ should limit the carrier mobility. Improvements in device fabrication, can lead to improved performance with respect to these values open promising prospects for optoelectronic applications.

## Results and Discussion

**Figure 1:**
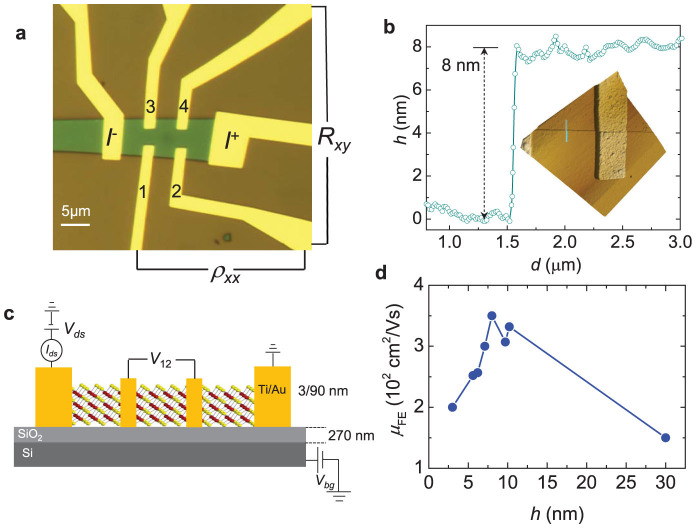
(a) Micrograph of the one of our WSe_2_ field-effect transistors on a 270 nm thick SiO_2_ layer on *p*-doped Si. Contacts, (Ti/Au) used to inject the electrical current (*I*_ds_), are indicated through labels *I*^+^ (source) and *I*^−^ (drain), while the resistivity of the device ρ*_xx_* was measured through either the pair of voltage contacts labeled as 1 and 2 or pair 3 and 4. The Hall resistance *R_xy_* was measured with an AC excitation either through the pair of contacts 1 and 3 or 2 and 4. Length l of the channel, or the separation between the current contacts, is l = 15.8 μm while the width of the channel is *w* = 7.7 μm. (b) Height profile (along the blue line shown in the inset) indicating a thickness of 80 Å, or approximately 12 atomic layers for the crystal in (a). Inset: atomic force microscopy image collected from a lateral edge of the WSe_2_ crystal in (a). (c) Side view sketch of our field-effect transistor(s), indicating that the Ti/Au pads contact all atomic layers, and of the experimental configuration of measurements. (d) Room temperature field-effect mobility μ_FE_ as a function of crystal thickness extracted from several FETs based on WSe_2_ exfoliated onto SiO_2_. The maximum mobility is observed for ~12 atomic layers.

Figures 1a and b show respectively, a micrograph of a typical device, whose experimental results will be discussed throughout this manuscript, and the sketch of a four-terminal configuration for conductance measurements. Current source *I*^+^ and drain *I*^−^ terminals, as well as the pairs of voltage contacts 1, 2 and 3, 4 are indicated. As shown below, this configuration of contacts allows us to compare electrical transport measurements performed when using a 2-contact configuration (e.g. μ_FE_) with a 4-terminal one (e.g. *R*_xy_ or the Hall-effect). Figure 1b shows an atomic force microscopy profile and image (inset) from which we extract a flake thickness of ~8 nm, or approximately 12 atomic layers. We chose to focus on multi-layered FETs because our *preliminary* observations agree with those of Refs. 19, 20, indicating that the highest mobilities are observed in flakes with thicknesses between ~10 and 15 atomic layers as shown in Fig. 1d. In addition, as argued in Ref. 5 multilayered flakes should lead to thin film transistors yielding higher drive currents when compared to transistors based on single atomic layers, possibly making multilayered FETs more suitable for high-resolution liquid crystal and organic light-emitting diode displays^5^. Our flakes were mechanically exfoliated and transferred onto a 270 nm thick SiO_2_ layer grown on *p*-doped Si, which is used as a back gate. Throughout this study, we focus on devices with thicknesses ranging from 9 to 15 layers. Three of the devices were annealed at 150 °C, under high vacuum for 24 h, which as reported in Ref. 17, yields higher mobilities particularly at low temperatures. We found very similar overall response among the non-annealed samples, as well as among the annealed ones.

**Figure 2:**
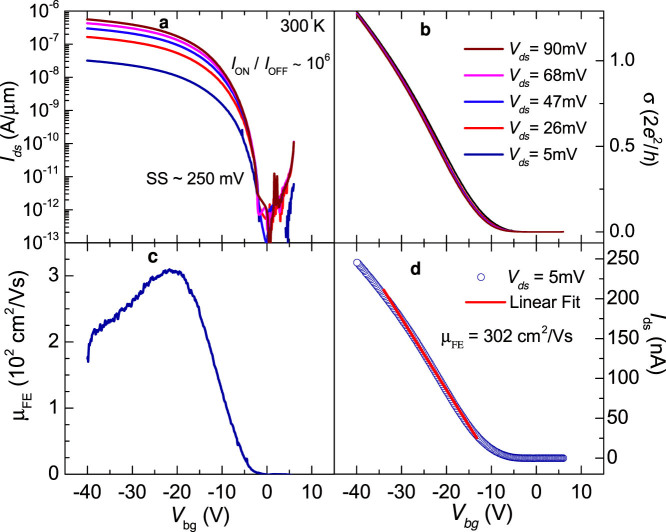
(a) Current *I*_ds_ in a logarithmic scale as extracted from a WSe_2_ FET at *T* = 300 K and as a function of the gate voltage *V*_bg_ for several values of the voltage *V*_ds_, i.e. respectively 5 (dark blue line), 26 (red), 47 (blue), 68 (magenta), and 90 mV (brown), between drain and source contacts. Notice that the ON/OFF ratio approaches 10^6^ and subthreshold swing SS ~250 mV per decade. We evaluated the resistance *R*_c_ of the contacts by performing also 4 terminal measurements (see Fig. 7 a below) through *R*_c_ = *V*_ds_/*I*_ds_ – ρ*_xx_* l/*w*, where ρ*_xx_* is the sheet resistivity of the channel measured in a four-terminal configuration. We found the ratio *R*_c_/ρ*_xx_* ≈ 20 to remain nearly constant as a function of *V*_bg_. (b) Conductivity σ = *S* l/*w*, where the conductance *S* = *I*_ds_/*V*_ds_ (from (a)), as a function of *V*_bg_ and for several values of *V*_ds_. Notice, how all the curves collapse on a single curve, indicating linear dependence on *V*_ds_. As argued below, this linear dependence most likely results from thermionic emission across the Schottky-barrier at the level of the contacts. (c) Field effect mobility μ_FE_ = (1/*c*_g_ *d*σ/*dV*_bg_ as a function of *V*_bg_, where *c*_g_ = ε_r_ε_0_/*d* = 12.789 × 10^−9^ F/cm^2^ (for a *d* = 270 nm thick SiO_2_ layer). (d) *I*_ds_ as a function of *V*_bg_, when using an excitation voltage *V*_ds_ = 5 mV. Red line is a linear fit whose slope yields a field-effect mobility μ_FE_ ≈ 300 cm^2^/Vs.

Figure 2a shows the extracted field-effect current *I*_ds_ as a function of the back gate voltage *V*_bg_ for several fixed values of the voltage *V*_ds_ across the current contacts, i.e. when using a 2-terminal configuration. From initial studies^7^, but in contrast with Refs. 25,26, WSe_2_ is expected to show ambipolar behavior, i.e. a sizable current resulting from the accumulation of either electrons or holes at the WSe_2_/SiO_2_ interface due to the electric field-effect. Although we have previously observed such a behavior, all FETs studied here show a rather modest electron current (i.e. saturating at ~10^−8^ A) at positive *V*_bg_ values in contrast also with samples covered with Al_2_O_3_, see Ref. 26. Therefore our samples behave as if hole-doped (i.e. sizeable currents only for negative gate voltages). At room temperature the minimum current is observed around *V*_bg_ ≈ 0 V while the difference in current between the transistor in its “ON”-state with respect to the OFF- one (on/off ratio) is >10^6^. For all measurements, the maximum channel current was limited in order to prevent damaging our FETs. The subthreshold swing SS is found to be ~250 mV per decade, or ~3.5 times larger than the smallest values extracted from Si MOSFETs at room temperature. Figure 2b shows the conductivity σ = *I*_ds_ l/*V*_ds_*w* (from a), as a function of *V*_bg_ for several values of *V*_ds_. As indicated in the caption of Fig. 1 the separation between the current contacts, is l = 15.8 μm while the width of the channel is *w* = 7.7 μm. As seen, all curves collapse on a single curve indicating linear behavior, despite the claimed role for Schottky barriers at the level of contacts^19^. See also the Supplemental Information section for linear current-voltage characteristics for the range of excitation voltages used. Figure 2c: the field-effect mobility μ_FE_ can be evaluated in the standard way by normalizing by the value of the gate capacitance (*c*_g_ = 12.789 × 10^−9^ F/cm^2^) the derivative of the conductivity with respect to *V*_bg_. As seen, μ_FE_ increases sharply above *V*_bg_ ≈ 2 V reaching a maximum of ~305 cm^2^/Vs at *V*_bg_ ~−20 V, decreasing again beyond this value. Alternatively, the mobility can be directly evaluated through the slope of *I*_ds_ as a function of *V*_bg_ in its linear regime, and by normalizing it by the sample geometrical factors, the excitation voltage *V*_bg_ and the gate capacitance *c*_g_, yielding a peak value μ_FE_ ≈ 302 cm^2^/Vs. We have observed μ_FE_ values as high as 350 cm^2^/Vs (see results for sample 2 below). These values, resulting from two-terminal measurements, are comparable to those previously reported by us for multi-layered MoS_2_, where we used a four-terminal configuration to eliminate the detrimental role played by the less than ideal contacts^27^.

**Figure 3:**
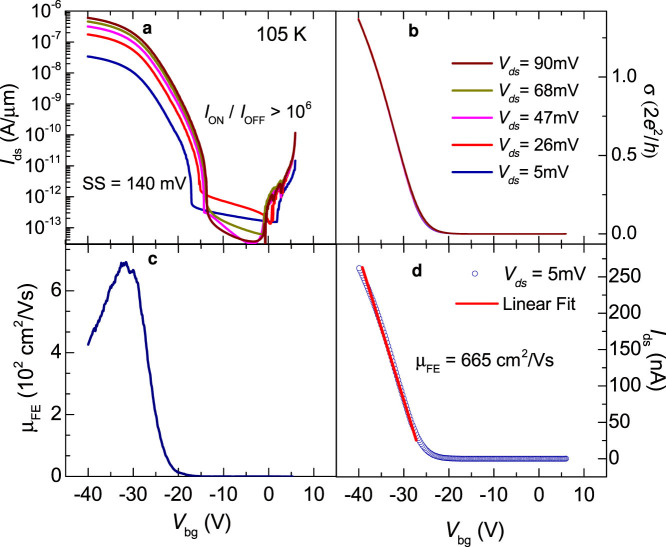
(a) Current *I*_ds_ in a logarithmic scale as extracted from the same WSe_2_ FET in Fig. 2 at *T* = 105 K and as a function of the gate voltage *V*_bg_ for several values of the voltage *V*_ds_, i.e. respectively 5 (dark blue line), 26 (red), 47 (magenta), 68 (dark yellow), and 90 mV (brown). Notice that the ON/OFF ratio still approaches 10^6^. (b) Conductivity σ as a function of *V*_bg_ for several values of *V*_ds_. Notice that even at lower *T*s all the curves collapse on a single curve. Notice how the threshold gate voltage *V^t^*_bg_ for conduction increases from ~0 V at 300 K to ~15 V at 105 K. Below, we argue that the observation of, and the increase of *V^t^*_bg_ as *T* is lowered, corresponds to evidence for charge localization within the channel. (c) Field effect mobility μ_FE_ = (1/*c*_g_) *d*σ/*dV*_bg_ as a function of *V*_bg_. (d) *I*_ds_ as a function of *V*_bg_, when using an excitation voltage *V*_ds_ = 5 mV. Red line is a linear fit whose slope yields a field-effect mobility μ_FE_ ≈ 665 cm^2^/Vs.

Figures 3a, b, c, and d show respectively, *I*_ds_ as a function of *V*_bg_ for several values of *V*_ds_, the corresponding conductivities σ as a function of *V*_bg_, and the resulting field-effect mobility as previously extracted through Figs. 2c and d. All curves were acquired at *T* = 105 K. As seen, at lower temperatures σ(*T*, *V*_bg_) still shows a linear dependence on *V*_ds_ although lower *T*s should be less favorable for thermally activated transport across Schottky barriers. In fact, we collected similarly linear data sets at *T* < 105 K. At *T* = 105 K, μ_FE_ displays considerably higher values, i.e. it surpasses 650 cm^2^/Vs (accompanied by a reduction in the SS down to ~140 mV per decade). However, as seen in Fig. 3a, lower temperatures increase the threshold gate voltage *V*^t^_bg_ required for carrier conduction. Below we argue that this is the result of a prominent role played by disorder and/or charge traps at the interface between WSe_2_ and SiO_2_ instead of just an effect associated with the Schottky barriers. Large Schottky barriers are expected to lead to non-linear current *I*_ds_ as a function of the excitation voltage *V*_ds_ characteristics, with a sizeable *I*_ds_ emerging only when *V*_ds_ surpasses a threshold value determined by the characteristic Schottky energy barrier ϕ, as seen for instance in Ref. 28. But according to Figs. 2b and 3b, σ is basically independent on *V*_ds_ above a threshold gate voltage, *even at lower temperatures*.

**Figure 4:**
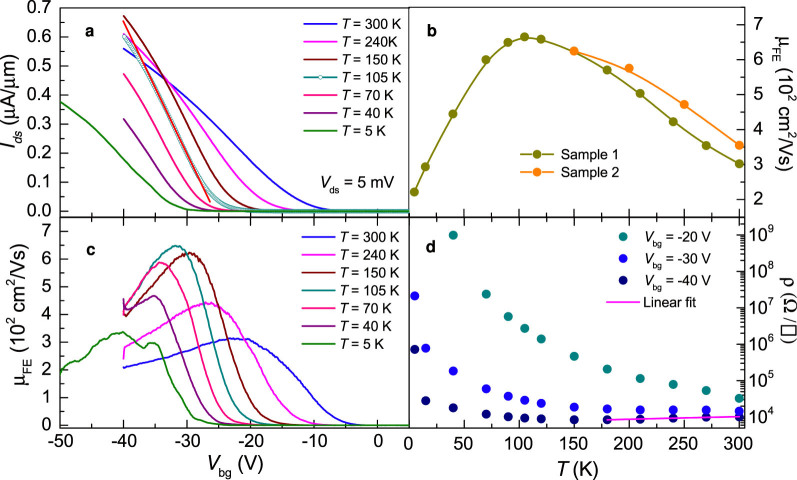
(a) *I*_ds_ as a function of the gate voltage *V*_bg_ for several temperatures *T* and for an excitation voltage *V*_ds_ = 5 mV. From the slopes of the linear fit (red line) one extracts the respective values of the field-effect mobility μ_FE_ as a function of the temperature, shown in (b). Orange markers depicts μ_FE_ for a second, annealed sample. The field-effect mobility is seen to increase continuously as the temperature is lowered down to *T* = 105 K, beyond which it decreases sharply. (c) μ_FE_ = (1/*c*_g_) *d*σ/*dV*_bg_ as extracted from the curves in (a). Notice that μ_FE_ still saturates at a value of ≈ 300 cm^2^/Vs at *T* = 5 K. d Resistivity ρ = 1/σ as a function of *T* for 3 values of the gate voltage, i.e. −20, −30 and −40 V, respectively (as extracted from the data in (a) or (c)). Magenta line corresponds to a linear fit, describing the behavior of the metallic resistivity, defined by ∂ρ/∂*T* > 0, observed at higher temperatures when *V*_bg_ = −40 V.

Figure 4a shows *I*_ds_ as a function of *V*_bg_ for several temperatures and for the crystal shown in Fig. 1a. Fig. 4b shows the resulting field-effect mobility μ_FE_ as a function of *T* as extracted from the slopes of *I*_ds_(*V*_bg_, *T*). μ_FE_ is observed to increase, reaching a maximum of ~650 cm^2^/Vs at *T* ~ 100 K, decreasing subsequently to values around 250 cm^2^/Vs at low temperatures. Orange markers depict μ_FE_ for a second, annealed sample whose Hall mobility is discussed below. This decrease is attributable to extrinsic factors, such as chemical residues from the lithographic process, since annealing the samples under high vacuum for at least 24 h considerably increases the mobility at low *T*s^17^, as will be illustrated by the results shown below for a second sample annealed in this way. Figure 4c shows μ_FE_ as a function of *V*_bg_ for several temperatures (as extracted from the curves in a). All curves show a maximum at a *V*_bg_-dependent value. As seen, the main effect of lowering *T* is to increase the threshold back-gate voltage *V*^t^_bg_ for carrier conduction. In WS_2_, by using ambipolar ionic liquid gating, which heavily screens charged defects, the authors of Ref. 29 were able to estimate the size of its semiconducting gap, given roughly by the difference between the threshold voltages required for hole and electron conduction respectively, or ~1.4 V. The much larger *V*^t^_bg_ values observed by us in WSe_2_ is attributable to intrinsic and extrinsic effects, such as vacancies and charge traps, which limit the carrier mobility becoming particularly relevant at low temperatures, see discussion below. At first glance, at low gate voltages ρ would seem to follow activated behavior with a small activation gap. On the other hand at high temperatures and high gate voltages, ρ displays metallic like behavior, usually defined by ∂ρ/∂*T* > 0. Magenta line is a fit to a simple linear-dependence on temperature, suggesting either an unconventional metallic state or most likely, phonon scattering.

**Figure 5:**
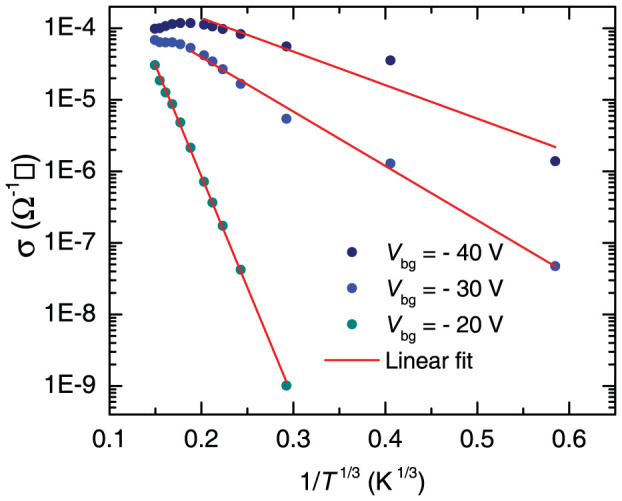
Conductivity, i.e. σ = 1/ρ (from the data in Fig. 4 d, acquired under *V*_ds_ = 5 mV) in a logarithmic scale as a function of *T*^−1/3^. Red lines are linear fits, indicating that at lower *T*s and for gate voltages below a temperature dependent threshold value *V*^t^_bg_(*T*), σ(T) follows the dependence expected for two-dimensional variable-range hopping.

As observed in Figs. 4a and c, the threshold gate-voltage *V*^t^_bg_ required to observe a finite σ increases from ~5 to ~35 V as *T* is lowered from 300 to 5 K. In order to clarify the dependence of *V*^t^_bg_ on *T*, we assume that *V*^t^_bg_ is dominated by disorder at the interface between WSe_2_ and SiO_2_ which leads to charge localization. To illustrate this point, in Fig. 5 we plot σ(*T*) as function of *T*^−1/3^ since from past experience on Si/SiO_2_ MOSFETs, it is well known that spurious charges intrinsic to the SiO_2_ layer^30^,^31^,^32^, in addition to the roughness at the interface between the Si and the glassy SiO_2_^33^, produces charge localization leading to variable-range hopping conductivity: σ(*T*) = σ_0_ exp(-*T*_0_/*T*)^1/(1+*d*)^ where *d* is the dimensionality of the system, or *d* = 2 in our case^34^. As seen in Fig. 5, one observes a crossover from metallic-like to a clear two-dimensional variable-range hopping (2DVRH) conductivity below a gate voltage dependent temperature; red lines are linear fits. At lower gate voltages, the 2DVRH regime is observed over the entire range of temperatures. Therefore, despite the linear transport regime and the relatively large mobilities observed in Figs. 1 through 4, this plot indicates very clearly, that below *V*^t^_bg_ the carriers in the channel are localized due to disorder. Notice that similar conclusions were also reported from measurements on MoS_2_^35^. Although, at the moment we do not have a clear experimental understanding on the type and on the concomitant role of disorder in these systems (which would allow a deeper theoretical understanding on the origin of the localization), the above experimental plot is unambiguous in revealing the predominant conduction mechanism for gate-voltages below a threshold value.

Now, we are in position of qualitatively explaining the *T*-dependence of *V*^t^_bg_: thermal activated processes promote carriers across a mobility edge which defines the boundary between extended electronic states and a tail in the density of states composed of localized electronic states. At higher temperatures, more carriers are thermally excited across the mobility edge, or equivalently, can be excited across the potential well(s) produced by disorder or charge traps, therefore one needs lower gate voltage(s) to untrap the carriers. Once these carriers have moved across the mobility edge, they become mobile and, as our results show, respond linearly as a function of the excitation voltage *V*_ds_. Finally, as *V*^t^_bg_ increases with decreasing *T* the number of carriers is expected to *decrease* continuously since they become progressively localized due to the suppression of thermally activated processes which can no longer contribute to carrier detrapping. This is clearly illustrated by Fig. 4b, where one sees an increase in mobility, due to the suppression of phonon scattering, leading to a maximum in the mobility and to its subsequent suppression upon additional cooling. Therefore, at higher temperatures and for gate voltages above the threshold, where one observes a metallic-like state, one has two competing mechanisms at play upon cooling, i.e. the tendency to localization/suppression of carriers which is unfavorable to metallicity, and the suppression of phonon scattering. Suppression of phonon scattering is the only possible explanation for the observed metallic behavior. Hence, one must conclude that this metallic behavior ought to be intrinsic to the compound, but disorder-induced carrier localization dominates σ at lower temperatures.

**Figure 6:**
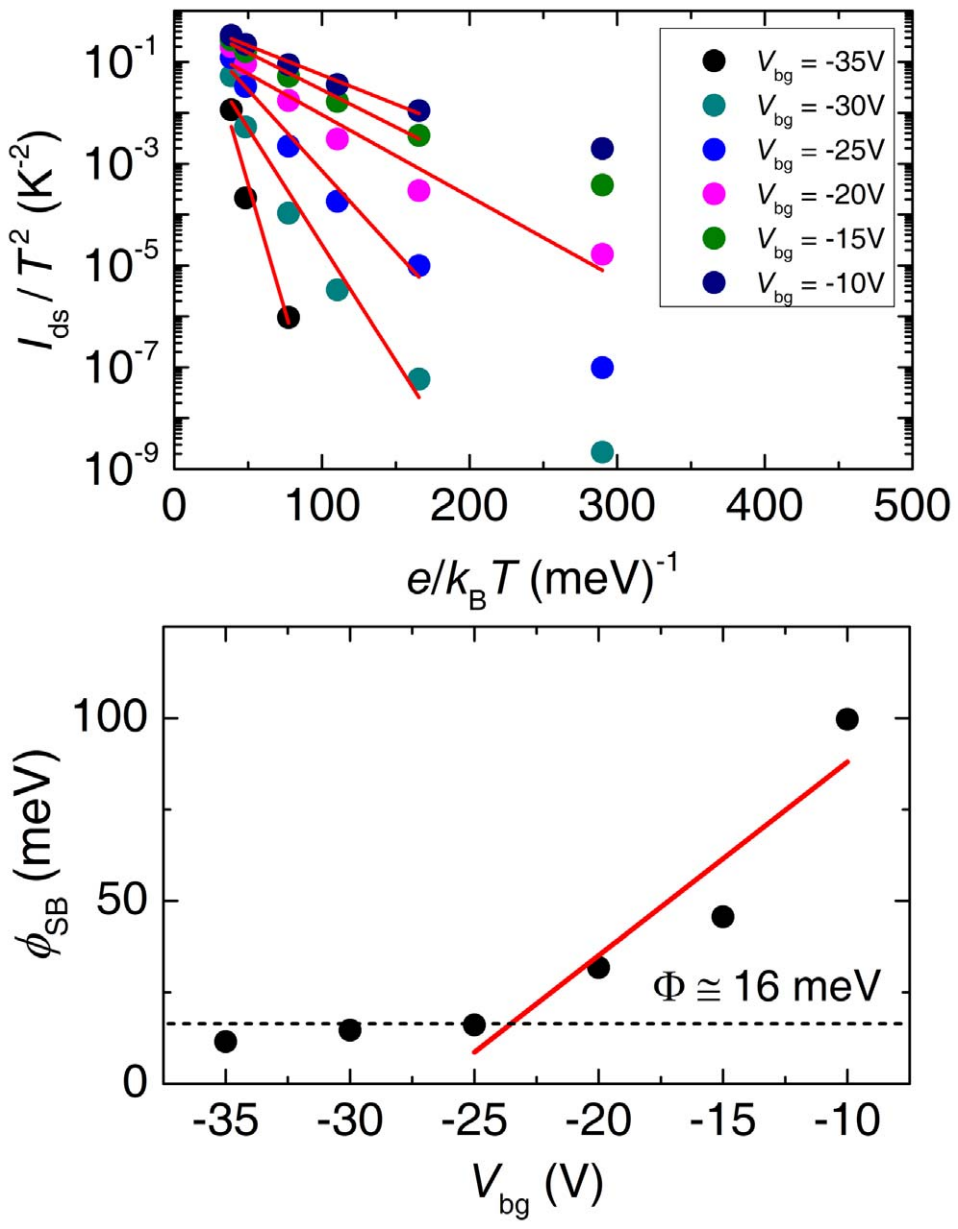
Top panel: Drain to source current *I*_ds_ as a function of (*k*_B_*T*/*e*)^−1^ for several values of the gate voltage *V*_bg_ (from the data in Fig. 4a). Red lines are linear fits from which we extract the value of the Schottky energy barrier ϕ_SB_. Bottom panel: ϕ_SB_ in a logarithmic scale as a function of *V*_bg_. Red line is a linear fit. The deviation from linearity indicates when the gate voltage matches the flat band condition^19^ from which we extract the size of the Schottky barrier Φ ≈ 16 meV.

Although, as Figs. 2 and 3 indicate, the conductivity σ as measured through a two-terminal configuration, is linear on excitation voltage *V*_ds_ when *V*_bg_ > *V*^t^_bg_, it was discussed at length that the electrical conduction through the drain and source contacts can by no means be ohmic^19^,^36^. In effect, a Schottky barrier of ~770 meV is expected as the difference in energy between the work function of Ti, or 4.33 eV, and the ionization energy of WSe_2_, or ~5.1 eV^37^,^38^. The linear, or apparent ohmic regime presumably would result from thermionic emission or thermionic field emission processes. According to thermionic emission theory, the drain-source current *I*_ds_ is related to the Schottky barrier height ϕ_SB_ through the expression:
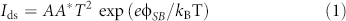
Where *A* is the area of the Schottky junction, A* = 4πem*k_B_^2^*h*^−3^ is the effective Richardson constant, *e* is the elementary charge, *k*_B_ is the Boltzmann constant, m* is the effective mass and *h* is the Planck constant^39^. In order to evaluate the Schottky barrier at the level of the contacts, in the top panel of Fig. 6 we plot *I*_ds_ normalized by the square of the temperature *T*^2^ as a function of *e*/*k*_B_*T* and for several values of the gate voltage. Red lines are linear fits from which we extract the ϕ_SB_(*V*_bg_). Notice that in the top panel of Fig. 6 the linear fits are limited to higher temperatures since at lower temperatures one observes pronounced, gate dependent, deviations from the thermionic emission theory. The bottom panel of Fig. 6 shows ϕ_SB_(*V*_bg_) in a logarithmic scale as a function of *V*_bg_. Red line is a linear fit from whose deviation we extract the size of the Schottky barrier^19^, or Φ ~16 meV, indicating a much better band alignment than originally expected. It is perhaps possible that the Eq. (1) might take a different form for layered two-dimensional materials, for example, in such compounds one might need a temperature pre-factor distinct from *T*^2^. We attempted the use of different temperature pre-factors such as *T* or *T*^3/2^, but it does not improve the linearity of log(*I*_ds_/*T*^α^) (with 2 ≥ α ≥ 1) as a function of *ek*_B_/*T*. In fact, an arbitrary *T* pre-factor, would not be theoretically justifiable at the moment. Having said that, one has to be very careful with the extraction of the Schottky barrier through this common approach, since the two-terminal measurements contain contributions from both the contacts and the conduction channel. As discussed above, the channel underdoes disorder-induced carrier localization, thus masking the true behavior of the conduction across the contacts. Notice for example, how in Fig. 5 2DVRH fits the behavior of the σ(*T*) over the entire range of temperatures when *V*_bg_ = −20 V, while in Fig. 6, thermionic emission can describe the behavior of *I*_ds_/*T*^2^ as a function of *T*^−1^ only when *T* > 125 K. Therefore the values of ϕ_SB_(*V*_bg_) extracted here should be taken with caution.

**Figure 7:**
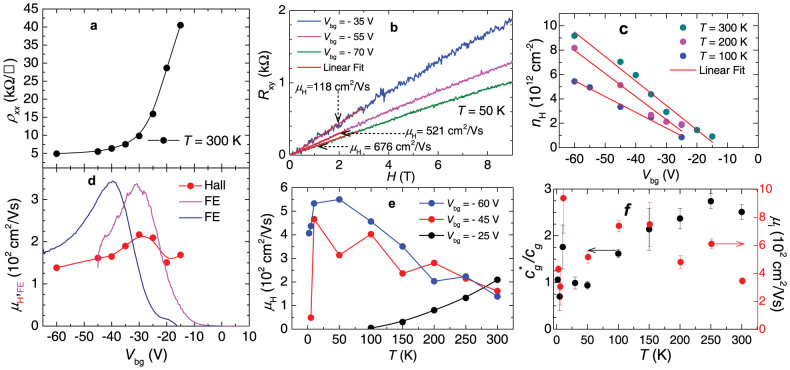
(a) Four-terminal sheet resistance *R_xx_* measured at a temperature of *T* = 300 K and as a function of *V*_bg_ for a second multilayered WSe_2_ FET after annealing it under vacuum for 24 h. (b) Hall response *R_xy_* = *V*_H_(*H*)/*I*_ds_ as a function of the external magnetic field *H*, and for several values of the gate voltage *V*_bg_. Red lines are linear fits from whose slope we extract the values of the Hall constant *R*_H_( = *V*_H_/*HI*_ds_). (c) Density of carriers *n*_H_ = 1/(e*R*_H_) induced by the back gate voltage as a function of *V*_bg_. Red lines are linear fits from which, by comparing the resulting slope *σ* = *n*/*V*_bg_ = *c*_g_*/*e* (*c*_g_* is the effective gate capacitance). (d) Field-effect μ_FE_ (magenta and blue lines) and Hall μ_H_ = *R*_H_/ρ*_xx_* (red markers) mobilities (where ρ*_xx_* = *R_xx_w*/l, *w* and l are the width and the length of the channel, respectively) as functions of *V*_bg_ at *T* = 300 K. (e) Extracted Hall mobility μ_H_ as a function of *T* and for several values of *V*_bg_. μ_H_ increases as *T* is lowered, but subsequently it is seen to decrease below a *V*_bg_ -dependent *T*. (f) Ratio between experimentally extracted and the ideal, or geometrical gate capacitances *c*_g_*/*c*_g_ (black markers) and the mobilities μ_i_ = *c*_g_*/*c*_g_ μ_H_ (*V*_bg_ = −60 V) (red markers) as functions of *T*. μ_i_ are the mobility values that one would obtain if the gate capacitance displayed its ideal *c*_g_ value in absence of spurious charges in the channel.

In Figure 7, we compare the above field-effect mobilities with Hall mobility measurements on a second, vacuum annealed flake of similar thickness. Figure 7 a shows the four-terminal sheet resistivity, i.e. ρ*_xx_* = *wV*_ds_/l*I*_ds_ as a function of *V*_bg_. ρ*_xx_* was measured with a lock-in technique, for gate voltages where the voltages *V*_12_ or V_34_ were in phase with the excitation signal. We also checked that any pair of voltage contacts produced nearly the same value for ρ*_xx_*, indicating a nearly uniform current throughout the channel. ρ*_xx_* increases very rapidly, beyond 10^9^ Ω as *V*_bg_ → 0 V. Also the out-of-phase component of the measured AC signal becomes very large as *V*_bg_ → 0 limiting the *V*_bg_ range for our measurements. Figure 7b displays the measured Hall signal *R*_xy_ as a function of the magnetic field *H* at *T* = 50 K and for several values of *V*_bg_. Red lines are linear fits from which we extract the Hall constant *R*_H_ = *R_xy_/H* = 1/*ne*. In the same Fig. 7b we also indicate the extracted values for the Hall mobilities, μ_H_ = *R*_H_/ρ*_xx_*, at different gate voltages. Notice that for *T* = 50 K and *V*_bg_ = 70 V one obtains, in this annealed sample, a μ_H_ value of ~676 cm^2^/Vs. Figure 7c shows the density of carriers *n*_H_ = 1/*eR*_H_ as a function of *V*_bg_ for several *T*s. Red lines are linear fits from which we extract the slope *n*_H_/*V*_bg_ = *c*_g_*/*e*, where *c*_g_* is an effective back-gate capacitance: in the absence of extrinsic charged defects at the WSe_2_/SiO_2_ interface, *c*_g_* should be equal to the previously quoted gate capacitance *c*_g_. Solid evidence for the existence of ionized impurities acting as hole traps at the interface is provided by the linear fits in Fig. 7c which intercepts the *n*_H_ = 0 axis at finite threshold gate voltages *V*^t^_bg_. This confirms that practically all holes generated by applying a gate voltage smaller than *V*^t^_bg_ remain localized at the interface. Figure 7d shows a comparison between μ_FE_ (magenta and blue lines) and μ_H_ (red markers) as extracted from the same device at room temperature. The blue line was measured after thermally cycling the FET down to low temperatures. Notice how *V*^t^_bg_ increases after thermally cycling the sample, thus suggesting that strain at the interface, resulting from the difference between the thermal expansion coefficients of SiO_2_ and WSe_2_, also contributes to *V*^t^_bg_. Therefore, strain would seem to be an additional factor contributing to the mobility edge. Notice also that both mobilities initially increase as a function |*V*_bg_|, reaching a maximum at the same *V*_bg_ value, decreasing subsequently as the back-gate voltage is further increased. Figure 7e shows μ_H_ as a function of *T* for several values of *V*_bg_. Notice how μ_H_ (*T* → 0 K) is suppressed at low gate voltages due to the charge localization mechanism discussed above. μ_H_ is observed to increase as *T* is lowered, requiring ever increasing values of *V*_bg_ > *V*^t^_bg_, but decreases again below *T* ~5 K. A fit of μ_H_(*T*, *V*_bg_ = −60 V) to *AT* ^−α^ yields α ~ (1 ± 0.1). Finally Fig. 7f displays the *T*-dependence of the ratio between the measured and the ideal geometrical gate capacitance (*c*_g_* = *se*)/*c*_g_ where *s* corresponds to the slopes extracted from the linear-fits in Fig. 7c. For a perfect FET this ratio should be equal to 1, i.e. the only charges in the conducting channel should be those resulting from the electric field-effect. Therefore, one can estimate the carrier mobility μ_i_ for the nearly ideal device, i.e. with the ideal geometrical capacitance, through μ_i_ = *c*_g_*/*c*_g_ μ_H_, which at *T* = 300 K would lead to *V*_bg_-dependent mobilities ranging from 350 up to 525 cm^2^/Vs. This rough estimate does not take into account scattering processes resulting from for example, other sources of disorder within the channel. In agreement with Ref. 40, this indicates that in our WSe_2_ FETs the main scattering mechanism limiting the carrier mobility are not phonons, but ionized impurities and disorder, or that phonon scattering would still allow mobilities approaching, and probably surpassing, 500 cm^2^/Vs at room temperature. In *p*-doped Si the hole-mobility is observed to saturate at a value of ~475 cm^2^/Vs for doping levels below ~10^17^ per cm^3^, while a doping concentration of 10^19^ per cm^3^ yields mobilities of ~200 cm^2^/Vs as observed here^8^. Therefore, our work indicates that if one was able to improve the FET fabrication protocols, by minimizing the disorder such as interface roughness, spurious ionized impurities and dangling bonds at the interface, WSe_2_ could match the performance of *p*-doped Si, thus becoming suitable for specific applications^5^ with the added advantage of miniaturization, since the starting point would be just a few atomic layers.

Notice that the μ_FE_ values extracted here at higher *T*s would be overestimated if one considers the value of the gate capacitance extracted from the Hall effect, i.e. it would be two to three times larger than the expected geometrical capacitance, thus implying 2 to 3 times smaller values for μ_FE_. A number of reports on TMDs^16^,^19^,^20^ suggest room temperature field-effect mobilities ranging from 300 to ~700 cm^2^/Vs for MoS_2_ based FETs subjected to “dielectric engineering”. However, taken together with the debate in Refs. 11, 12 concerning the true value of the gate capacitance in dual gated FETs, our study suggests that those values should be carefully re-examined by performing four-terminal Hall-mobility and/or capacitance measurements.

In the Supplemental Information, we show the Raman spectra of WSe_2_ whose main Raman modes are observed to sharpen considerably as the number of layers decrease, implying a pronounced increase in phonon lifetimes. Possibly, the main source of disorder in WSe_2_ is stacking disorder, which is progressively eliminated as one decreases the number of layers. This also implies a high degree of in-plane crystallinity. On the other hand, polarized Raman indicates that most Raman modes in WSe_2_ are mixed modes, i.e. composed of in-plane and out-of-plane lattice vibrations, which might affect the strength of its electron-phonon coupling.

Although a gate-voltage dependent Raman study has yet to be performed in WSe_2_, in both single-layer^41^ and bi-layer^42^ graphene, it was observed that the gate-voltage can tune the interaction between phonons and the charge carriers, leading to changes in the amplitude and in the line-width of the Raman spectra. A similar gate-voltage dependence in WSe_2_ might reveal reduced electron-phonon scattering therefore explaining the higher room-temperature Hall mobilities observed here. Notice, that monolayer TMDs have been predicted to display strong piezoelectricity^43^, suggesting that these materials are prone to a strong coupling between lattice degrees of freedom and an external electric field.

## Conclusions

In summary field-effect transistors based on multi-layered *p*-doped WSe_2_ can display peak hole Hall-mobilities in excess of 200 cm^2^/Vs at room temperature. This value increases by a factor >3.3 when the temperature decreases to ~100 K. The carrier density as a function of the gate voltage, as extracted from the Hall-effect, indicates larger than expected gate capacitances thus implying an excess of spurious charges in the channel. Therefore, one should be cautious when quoting values for the field-effect mobility by using the geometrical gate capacitance value. These spurious charges, in addition to disorder at the WSe_2_/SiO_2_ interface, leads to carrier localization and to a concomitant mobility edge, which manifests itself in an increasing threshold gate voltage for carrier conduction and, at a fixed gate voltage, to a concomitant decrease in carrier mobility upon cooling (resulting from an increase in the threshold gate voltage). When using Ti:Au for the electrical contacts one obtains a remarkably small value for the size of the Schottky barrier, although thermionic emission theory can only properly fit the transport data at higher temperatures.

We emphasize that our results indicate that WSe_2_ displays what seemingly are the highest Hall mobilities observed so far in TMDs, particularly among FETs based on few-layered TMDs exfoliated onto SiO_2_ and remarkably, without the use of distinct or additional dielectric layers. The Hall mobility values observed here surpass, for example, the μ_H_ values in Ref. 17 for MoS_2_ on HfO_2_ or the field-effect mobilities of thicker multilayered MoS_2_ flakes^5^ on Al_2_O_3_. This indicates that WSe_2_ has the potential to display even higher carrier mobilities, particularly at room temperature, through the identification of suitable substrates (flatter interfaces, absence of impurities and dangling bonds, etc), as well as contact materials. A major materials research effort must be undertaken to clarify the density of point defects (e.g. vacancies, intercalants) in the currently available material and on how to decrease their density. However, our study reveals that WSe_2_ has the potential to become as good if not a better material for optoelectronic applications than, for instance, multi-layered MoS_2_^5^. Recently, Ref. 44 reported the performance of multi-layered WSe_2_ FETs, composed of WSe_2_ atomic layers transferred onto a *h*-BN substrate using graphene for the electrical contacts as well as ionic liquid gating. Remarkably, despite the complexity of this architecture, originally intended to improve the overall performance of multi-layered WSe_2_ FETs, the simpler devices reported here, still display considerably higher mobilities. We believe this is an important piece of information for those considering the development of electronic or optoelectronic applications based on transition metal dichalcogenides.

## Methods

WSe_2_ single crystals were synthesized through a chemical vapor transport technique using iodine as the transport agent. Multi-layered flakes of WSe_2_ were exfoliated from these single crystals by using the “scotch-tape" micromechanical cleavage technique, and transferred onto *p*-doped Si wafers covered with a 270 nm thick layer of SiO_2_. Prior to transferring the WSe_2_ crystals onto the SiO_2_ layers, these were cleaned in the following way: SiO_2_ was sonicated for 15 min in acetone, isopropanol and deionized water, respectively. It was subsequently dried by a nitrogen gas flow. For making the electrical contacts 90 nm of Au was deposited onto a 4 nm layer of Ti *via* e-beam evaporation. Contacts were patterned using standard e-beam lithography techniques. After gold deposition, the devices were annealed at 200°C for ~2 h in forming gas. Atomic force microscopy (AFM) imaging was performed using the Asylum Research MFP-3D AFM. Electrical characterization was performed by using a combination of sourcemeter (Keithley 2612 A), Lock-In amplifier (Signal Recovery 7265) and resistance bridges (Lakeshore 370) coupled to a Physical Property Measurement System. The Raman spectra were measured in a backscattering geometry using a 532.1 nm laser excitation. For additional details see the Supplementary Information.

## Author Contributions

L.B. conceived the project in discussions with N.R.P., S.T., M.T. and P.M.A. D.R. synthesized the WSe_2_ single crystals. N.R.P. and D.R. characterized the thickness of the used flakes though AFM techniques. N.R.P. fabricated the field-effect transistors. J.M.P., D.S. and M.T. performed polarized Raman experiments and their dependence on number of layers as well as the corresponding analysis. S.F., N.P.L., A.L.E. and M.T. have performed Raman measurements as a function of excitation frequency. N.R.P., S.M. and L.B. performed the electrical transport characterization. N.R.P. and L.B. analyzed the corresponding data. L.B. wrote the manuscript with the input of all co-authors.

## Supplementary Material

Supplementary Information Supplementary Information
