# Supplementary material for: Hall and field-effect mobilities in few layered p-WSe2 field-effect transistors
Source: Sci Rep. 2015 Mar 11;5:8979. doi: 10.1038/srep08979 (PMC4355631; doi:10.1038/srep08979)
Supplement: Supplementary Information [file srep08979-s1.pdf]

Supplemental information to manuscript titled: **"Hall and field-effect mobilities in few layered *p*-WSe_2 field-effect transistors"** by Nihar R. Pradhan[1], Daniel Rhodes[1], Shariar Memaran[1], Jean M. Poumirol[1], Dmitry Smirnov[1], Saikat Talapatra,[2] Simin Feng, Nestor Perea-Lopez,[3] Ana L. Elias,[4] Mauricio Terrones,[4] Pulickel M. Ajayan,[5] and Luis Balicas[1]

[1]National High Magnetic Field Laboratory, Florida State University, Tallahassee-FL 32310, USA
[2]Physics Department, Southern Illinois University, Carbondale-IL 62901-4401, USA
[3]Department of Physics, Department of Materials Science and Engineering and Materials Research Institute, The Pennsylvania State University, University Park, PA 16802, USA
[4]Department of Physics, Department of Materials Science and Engineering and Materials Research Institute. The Pennsylvania State University, University Park, PA 16802, USA
[5]Department of Mechanical Engineering and Materials Science, Rice University, Houston, TX 77005 USA

Current-Voltage characteristics and leakage voltage

In the right panel of Fig. S1 below, we show the current flowing through the drain-source contacts as a function of the excitation voltage V_{ds} for several values of the back gate voltage. As already inferred from Figs. 2 and 3 in the main text the response of our field-effect transistors is quite linear (as if ohmic) for excitation voltages below 100 mV. The left panel shows an example of the leakage current flowing through the back gate as the back gate voltage (in Fig. 2 within the main text) is swept. As seen, when the current through our FETs surpasses 1 μA, e.g. for $V_{bg} > 40$ V, the leakage current does not even reach 1 nA.

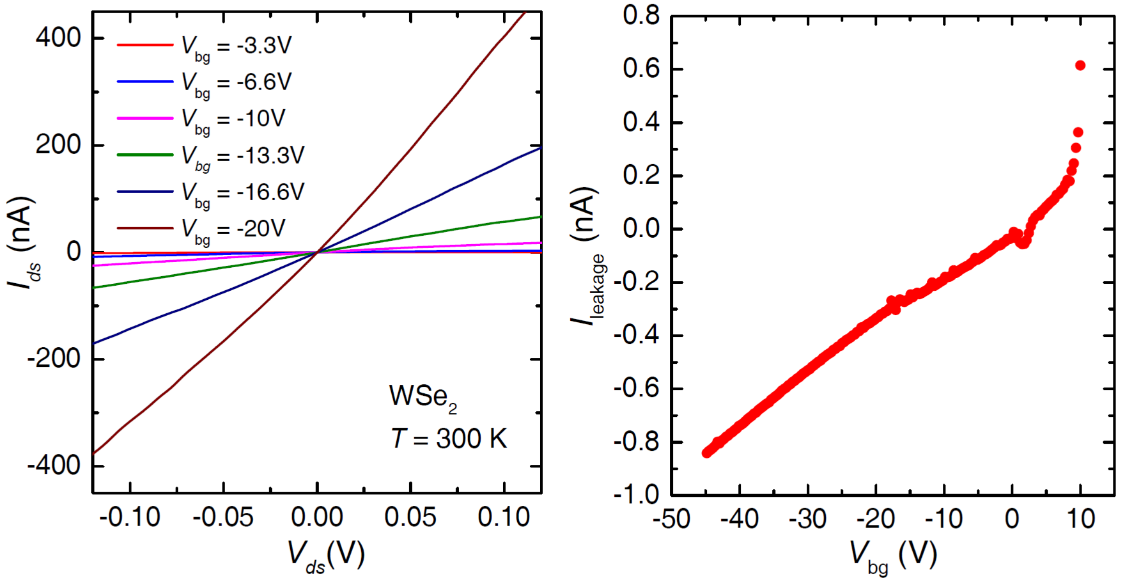

Figure S1| Left panel: Drain-source current I_{ds} as a function of the drain-source excitation voltage V_{ds} for several values of the back-gate voltage V_{bg}, at room temperature. Right panel: typical leakage current flowing through the back gate as function of the back gate voltage V_{bg} for one of our WSe_2 based FETs.

Evaluating the Schottky barriers through thermionic emission theory

When evaluating the Schottky barrier through thermionic emission theory, or through the expression (1) in the main text, i.e. $I_{ds} = AA^*T^2 \exp(e\phi_{SB}/k_BT)$, we have considered the

possibility that the low dimensionality of this system might lead to a power in temperature distinct to a T^2 dependence, such as a commonly assumed $T^{3/2}$ term, or even the possibility of a hitherto not reported T-linear pre-factor. As seen in Fig. S2 below, all three exponents on temperature lead to similar linear fits at higher temperatures. Hence, we cannot unambiguously define the correct power law. One could have left the exponent as a free fitting parameter. However, it would have been difficult to justify theoretically an arbitrary power law.

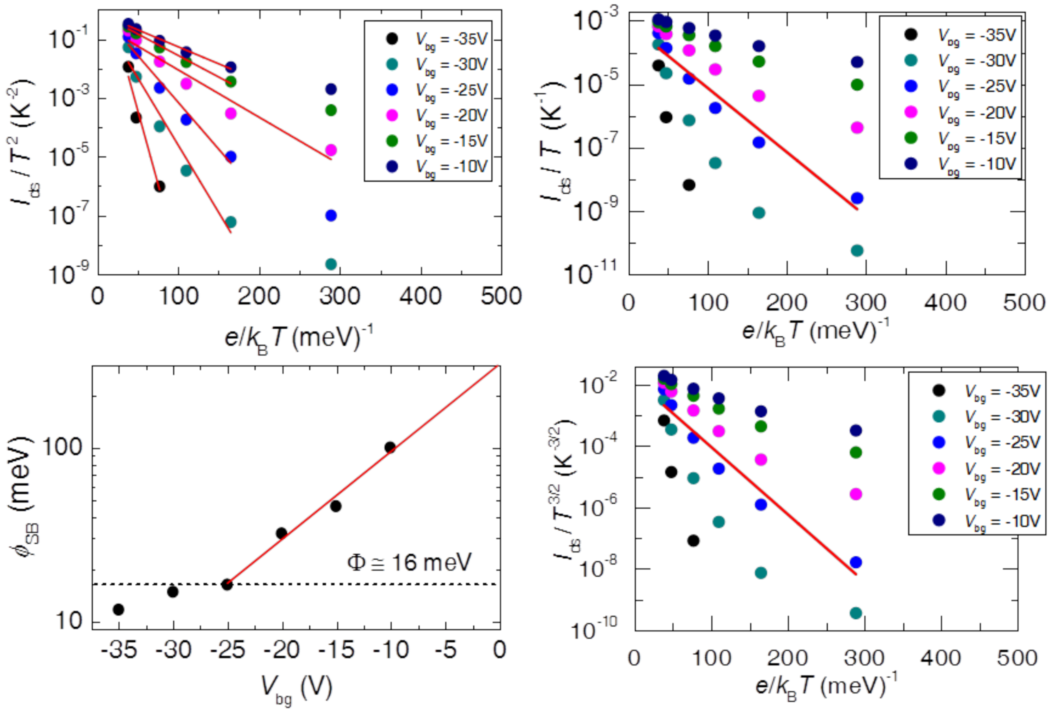

Figure S2| Top left panel: Drain to source current I_{ds} as a function of $(k_B T/e)^{-1}$ for several values of the gate voltage V_{bg} (from the data in Fig. 4 **a** in the main text). Red lines are linear fits from which we extract the value of the Schottky energy barrier ϕ_{SB}. Bottom left panel: ϕ_{SB} in a logarithmic scale as a function of V_{bg}. Red line is a linear fit. The deviation from linearity would indicate when the gate voltage matches the flat band condition[19] from which one would extract the size of the Schottky barrier $\Phi \approx 16$ meV. Top Right panel: I_{ds} *normalized by temperature* as a function of $(k_B T/e)^{-1}$. Bottom Right panel: I_{ds} normalized by $T^{3/2}$ as a function of $(k_B T/e)^{-1}$. In both panels the red lines are linear fits. Notice how the distinct power laws in temperature lead to linear fits of similar quality.

Raman Scattering

In order to understand the extremely high values of mobility shown here, we explored through Raman spectroscopy the quality of the flakes extracted from our single crystals. Here, our goal is to evaluate the width of the Raman peaks since it reflects the coherence and the mean free path of the phonons and therefore the strength of the electron-phonon scattering. A complete Raman study as a function of the number of layers, and laser frequency will be presented elsewhere.

The Raman spectra were measured in a backscattering geometry using a 532.1 nm laser excitation. The laser light was injected into an optical fiber, guiding the excitation to the sample

stage. The excitation spot size was about 10 mm in diameter. The scattered light collected by a x100 microscope objective and directed into a collection fiber, and then guided to a spectrometer equipped with a liquid-nitrogen-cooled CCD camera. The spectra were acquired in the spectral region from 150 to 330 cm^{-1} with a spectral resolution of approximately 1 cm^{-1}. The peak widths were obtained after correcting for instrumental broadening following the procedure in Ref. [S1]. The Raman spectra shown in Fig. S3 was acquired with an incident laser power of 1.5 mW; we observed that the Raman peaks (position and broadening) are insensitive to the power level when measured with laser power below power densities of 1500 W/cm^2.

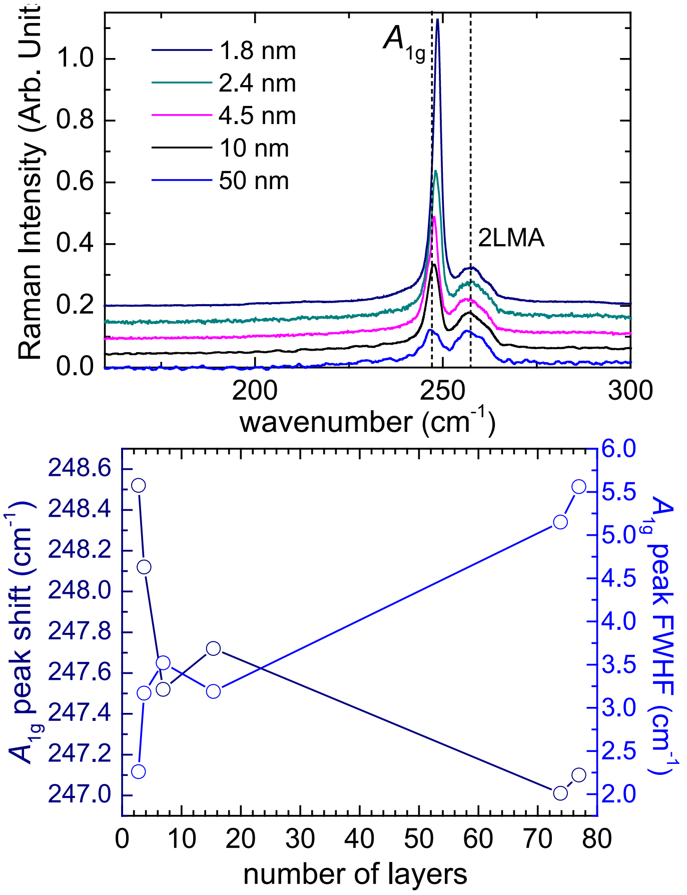

Figure S3| Top panel: Intensity of the Raman lines as a function of the wavenumber for several exfoliated WSe_2 flakes of distinct thicknesses. The thickness of each flake was determined through atomic force microscopy measurements. Notice how the peak associated with a mixed E_g+A_{1g} mode grows and sharpens as the number of layers is reduced. Lower panel: Position of the aforementioned peak as determined from Lorentzian fits as a function of the number of layers (dark blue markers). Blue markers depict the full width at half maximum (FWHM) of the E_g+A_{1g} peak as a function of the number of layers as resulting from the Lorentzian fits. Notice the shift to higher frequencies and the remarkable increase in sharpness as the number of layers decreases.

Here, we concentrate on the two main peaks observed in the range between 200 cm^{-1} and 300^{-1} when using a laser excitation of 532.1 nm. In a number of previous reports the leftmost peak was identified with the E^1_{2g} shear mode (vibration of the W-Se bond), and the broad feature at its right with the A_{1g} mode (out-of-plane optical vibration of the Se atoms). The A_{1g} mode is known to be sensitive to the polarization of the reflected light relative to the polarization of the incident beam while the E^1_{2g} is not. Through a polarized Raman scattering study we found the leftmost peak to be very sensitive to the relative polarization between the incident and the reflected light. However, it does not become completely inactive under cross-polarization indicating that it corresponds to a mode of mixed (E + A_{1g}) character. In effect, in monolayered WSe_2, the experimental Raman spectrum exhibits the presence of the perpendicular mode A'_1 and the in plane E' almost degenerate at around 250 cm^{-1} [S2, S3]; according to our calculations A'_1 is at 250.23 cm^{-1} and the E' at 249.36 cm^{-1} (for details, please see Ref. [S4]). By adding

layers, we observed that the out of plane modes A'_1 (A_{1g}) shift to higher frequencies and the E' (E_g) displace to lower frequencies, a behavior that has been reported experimentally by different authors in this and other STMDs (see, Fig. S3 above and also Refs. [S1-S5]). Experimentally, these modes in the bulk 3-D crystals are associated with the A_{1g} (located at 251 cm^{-1}) and the E_{2g} (around 247 cm^{-1}) irreducible representations of the D_{6h} point group, exhibiting inversion symmetry. In fact, for a larger number of layers we collected a rather complex Raman spectrum for WSe_2 with several additional higher-order mixed modes. Currently, we are performing additional calculations and measurements to understand this complex phonon spectrum which is similar to data collected by other groups [6], but at first glance looks *distinct* from the MoS_2 one [7].

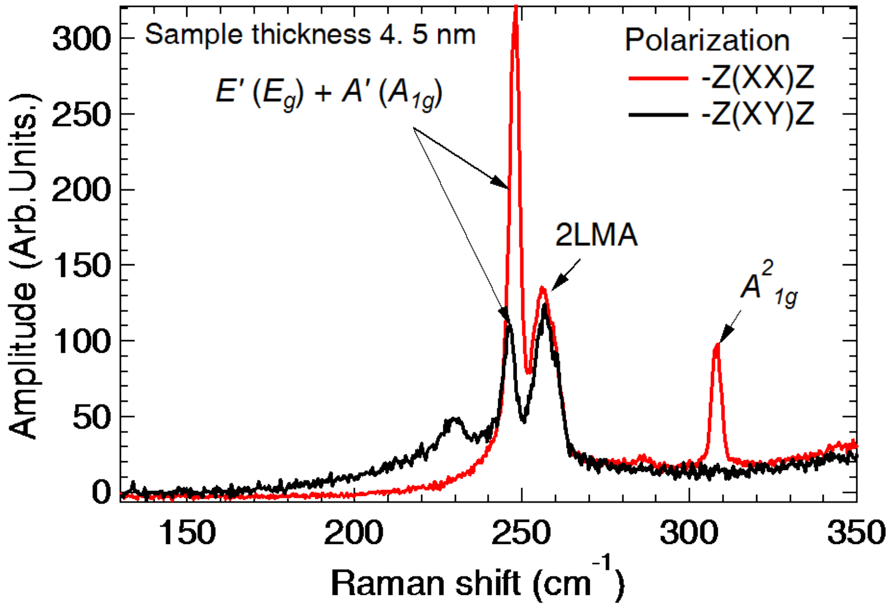

Figure S4| Cross polarized Raman spectra for 4.5 nm thick WSe_2 flake. Notice how a sharp peak becomes undetectable under cross polarization indicating that it corresponds to out-of-plane lattice vibration modes. According to the calculations in Ref. [S4] this peak corresponds to the A^2_{1g} mode. Finally, notice how the most pronounced peaks are suppressed, although not entirely by the cross polarization, suggesting that they correspond to mixed in-plane (E' or E_g) and out of plane modes (A' or A_{1g}). We indexed them based on the calculations of Ref. [S4].

In Fig. S3 we show the Raman spectra as a function of the wavenumber as extracted from several flakes of varying thicknesses. The thickness of the number of layers was determined through AFM measurements. As seen in Fig. 2 the mixed E' (E_g) + A' (A_{1g}) mode red shifts to higher wave-numbers as the number of layers decrease, as predicted and observed for the E^1_{2g} mode in MoS_2 [S7, S8, S9]. Remarkably, this shift starts in multi-layered flakes and becomes quite pronounced as the number of layers decrease. Most importantly and as seen in Fig. S3, the height of this E' (E_g) + A' (A_{1g}) peak increases considerably as the number of layers decrease. It also sharpens, i.e. by a factor greater than 2 with its full width at half maximum (FWHM) decreasing to a very small value of $\cong$ 2.2 cm^{-1} indicating a high in-plane crystallinity for the flakes which does not deteriorate as the number of layers decreases. On the contrary, it suggests an increase in phonon coherence as the number of layers is reduced due to a higher level of crystallinity, possibly due to the removal of defects such as stacking faults or stacking

disorder. Most importantly, a considerably large phonon coherence-length necessarily implies small electron-phonon scattering or very weak electron-phonon coupling. This might be an ingredient contributing to the larger mobilities observed here for the WSe_2 field-effect transistors. The height and position of the broad 2LMA mode is independent on the number of layers further indicating that the crystallinity of the flakes does not decrease with decreasing the number of layers.

In Fig. S4 above, we demonstrate that the most prominent Raman modes in WSe_2 are mixed modes, i.e. composed of both in-plane and out-of-plane vibrational modes. We speculate that the nature of the electron/hole-phonon scattering in WSe_2 might be somewhat distinct from the other transition metal dichalcogenides, explaining perhaps its higher carrier mobility at room temperature. This might also lead to a more pronounced effect of the gate voltage on the electron phonon coupling.

References

[S1] Tanabe, K., & Hiraishi, J. Experimental-determination of true Raman linewidths from measurements of linewidths observed at different slit openings. *Appl. Spectrosc.* **1981** 35, 436.

[S2] Zhao, W. , Ghorannevis, Z., Amara, K. K., Pang, J. R. , Toh, M. , Zhang, X., Kloc, C., Tan, P. H. and Eda, G. Lattice dynamics in mono- and few-layer sheets of WS_2 and WSe_2. *Nanoscale* **2013,** 5, 9677-9683.

[S3] Tonndorf, P , Schmidt, R., Bottger, P., Zhang, X., Borner, J., Liebig, A., Albrecht, M., Kloc, C., Gordan, O., Zahn, D. R. T., de Vasconcellos, S. M., Bratschitsch, R. Photoluminescence emission and Raman response of monolayer MoS_2, $MoSe_2$, and WSe_2. *Opt. Express* **2013**, 1, 4908-4916.

[S4] Terrones, H., Del Corro-Garcia, E., Feng, S., Poumirol, J. M., Smirnov, D., Rhodes, D. Pradhan, N.R., Zhong, L., Nguyen, M.A.T., Elías, A. L., Mallouk, T. E., Balicas, L., Pimenta, M., Terrones, M. New First Order Raman Active Modes in Few Layered Transition Metal Dichalcogenides. *Sci. Rep.* **2014**, 4, 4215.

[S5] Zhang, X., Han, W. P., Wu, J. B., Milana, S. , Lu, Y., Li, Q. Q., Ferrari, A. C. & Tan, P. H. Raman spectroscopy of shear and layer breathing modes in multilayer MoS_2, *Phys. Rev. B* **2013**, 87, 115413.

[S6] Li, H, Lu, G., Wang, Y., Yin, Z., Cong, C., He, Q., Wang, L., Ding, F., Yu, T., & Zhang, H., Mechanical Exfoliation and Characterization of Single- and Few-Layer Nanosheets of WSe_2, TaS_2, and $TaSe_2$. *Small* **2013**, 9, 1974–1981.

[S7] Lee, C., Yan, H., Brus, L. E., Heinz, T. F., Hone J., & Ryu, S. Anomalous Lattice Vibrations of Single- and Few-Layer MoS_2. *ACS Nano* **2010**, 4, 2695.

[S8] Zeng, H., Zhu, B., Liu, K., Fan, J., Cui, X., & Zhang, Q. M. Low-frequency Raman modes and electronic excitations in atomically thin MoS_2 films, *Phys. Rev. B* **2012**, 6, 241301(R).

[S9] Molina-Sanchez, A. & Wirtz, L. Phonons in single-layer and few-layer MoS_2 and WS_2. *Phys. Rev B* **2011**, 84, 155413.
